# Components Essential for the Generation of Germinal Centers

**DOI:** 10.1155/1998/47168

**Published:** 1998

**Authors:** L. V. Rizzo, E. A. Secord, V. K. Tsiagbe, D. T. Umetsu, R. H. Dekruyff, W. J. Simmons, G. J. Thorbecke

**Affiliations:** 1 Laboratory of Immunology Clinical Immunology Section NEI, NIH Bethesda MD 20892 USA; 2 Department of Pathology and Kaplan Cancer Center NYU School of Medicine New York NY 10016 USA; 3 Department of Pediatrics Stanford University Palo Alto CA 94305 USA; 4 New York University Medical Center 550 First Avenue New York NY 10016 USA


*Developmental Immunology*, 1998, Vol. 6, pp. 325–330
Reprints available directly from the publisher
Photocopying permitted by license only

© 1998 OPA (Overseas Publishers Association)
N.V. Published by license under the
Harwood Academic Publishers imprint,
part of the Gordon and Breach Publishing Group
Printed in Malaysia

### Components Essential for the Generation of Germinal Centers

L.V. RIZZO[a], E.A. SECORD[b], V.K. TSIAGBE[b], D.T. UMETSU[c], R.H. DEKRUYFF[c], W.J. SIMMONS[b] and G.J.THORBECKE[b]*

[a]*Laboratory of Immunology, Clinical Immunology Section, NEI, NIH, Bethesda, MD 20892;* [b]*Department of Pathology and Kaplan Cancer Center, NYU School of Medicine, New York, NY 10016;* [c]*Department of Pediatrics, Stanford University, Palo Alto, CA 94305*

The present discussion reviews the minimal requirements for germinal center (GC) production. Among the components needed to produce these typical *in vivo* structures are (1) antigen (Ag with epitopes recognized by T cells), (2) B cells specific for the Ag, and (3) CD4$^+$ T cells.

The fact that Ag is a determining factor in the induction of GCs has been clear since the observation that GCs are absent or very much reduced in germ-free guinea pigs (Glimstedt, 1936), chickens (Thorbecke et al., 1957), and rats (Thorbecke, 1959). Particularly striking is the enormously reduced number of GCs in gut-associated lymphoid tissue in the germ-free chicken (Thorbecke et al., 1957). However, an important question concerning the need for Ag is whether it has to be presented on follicular dendritic cells (FDC) for the initial induction of GCs or whether other dendritic cells and/or activated B cells themselves can serve as APCs on this occasion.

An interesting model that could be used to evaluate this question is presented by murine mammary tumor virus (MMTV) LTR-encoded superantigens (vSAgs), which are only expressed on the surface of cells, primarily on B cells. The lymphomas of SJL mice express such a vSAg, encoded by *Mtv29*. These lymphomas are GC-derived and totally dependent for their growth on the V$\beta$16$^+$CD4$^+$ T cells that their vSAg stimulates (Tsiagbe et al., 1993). The early lymphomas are clearly PNA$^+$, as may be seen in sections of (grossly normal-looking) Peyer's patches from occasional 6- 12-month-old SJL mice (Fig. 1). In similarly aged SJL mice bearing a *Bcl-2* transgene (targeted to B cells), there appear abnormal GCs as well as hyperplasia of normal-looking GCs, even in the medullary cords of lymph nodes where FDCs are not expected to be present (Secord et al., 1995; Ponzio et al., 1996). This form of GC hyperplasia is not seen in similarly aged BALB/c mice bearing the same *Bcl-2* transgene. We have interpreted these findings to indicate that there is synergy between the expression of Mtv29-vSAg on the surface of GC cells and a prolonged survival of GC cells due to enhanced BCL-2 expression. However, this interpretation is based on the presumption that surface presentation of vSAg by B cells can lead to GC formation.

Another interesting aspect of the role of Ag in GC formation is that persisting Ag or immune complex on FDCs (long after the initial primary response) by

*Corresponding author. Present address: New York University Medical Center, 550 First Avenue, New York, NY 10016.

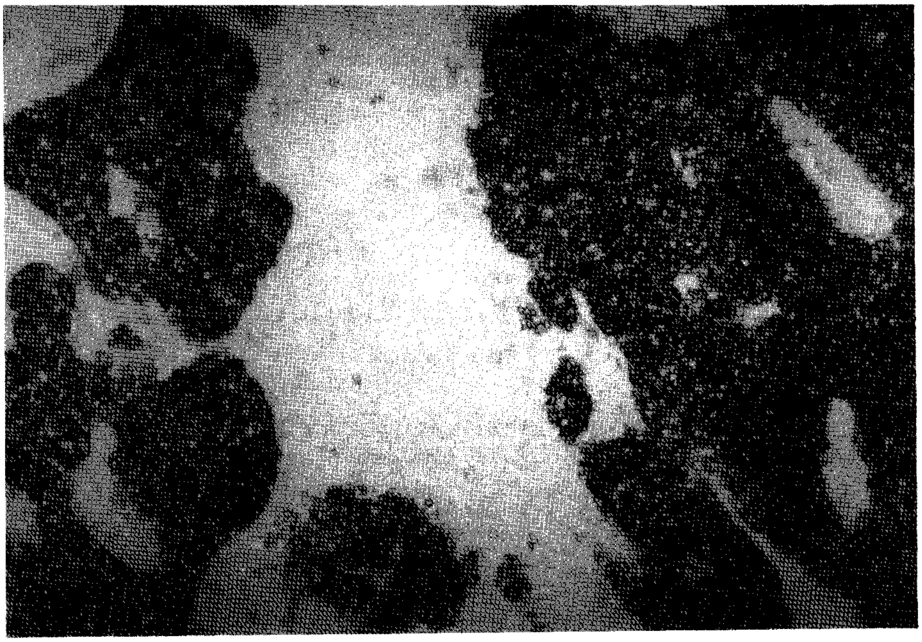

FIGURE 1 Abnormal germinal centers in Peyer's patch of middle-aged SJL mouse. Note the irregularly shaped areas of $PNA^+$ large blast cells in the lower part of the mucosa of this Peyer's patch. Staining was with peoxidase-labeled PNA and methyl green. Magnification: 80×.

itself, although apparently capable of influencing the fate of circulating (resting) memory B cells, does not cause GC formation unless renewed activation of T cells is induced (Baine et al., 1981).

With respect to the need for B cells, an important unresolved question remains: Is there a subset of B cells that is particularly good at giving rise to GCs? In previous work performed in collaboration with Linton and Klinman (Linton et al., 1992), we have shown that unprimed $HSA^{lo}$ ($J11D^{lo}$) B cells are much more proficient at producing GCs in SCID recipients in the presence of excess-carrier-primed helper T cells ($2 \times 10^6$) than are $HSA^{hi}$ B cells. As few as $10^5$ transferred $J11D^{10}$ BALB/c B cells gave rise to 9.4 GCs per spleen section within 7 days after cell transfer, as compared to 0.2 GC per spleen section in recipients of an equivalent number of $J11D^{hi}$ B cells.

It should be noted that the data from these cell transfer studies also suggest that functionally developed FDCs are not required for GC formation, since SCID mice are not reconstituted to exhibit such FDCs within a week after B- and T-cell transfer (Kapasi et al., 1993). The $HSA^{lo}$ GC precursor cells are also $IgD^{hi}$, which is in line with other findings from our laboratory in which we have found that the presence of receptors for IgD on T cells, induced by injection of oligomeric IgD, facilitates both GC production (Swenson et al., 1988) and the induction of early memory for antibody responses (Coico et al., 1983). Thus, the presence of $IgD^{hi}$ on B cells might facilitate T-B cell interaction leading to GC formation. Indeed, the augmenting effect of IgD-R expression on T cells is not observed in $IgD^{-/-}$ mice (Swenson et al., 1995). Moreover, the intravenous injection of monomeric IgD, which cannot cross-link IgD-R, prevents any immunoaugmenting or IgD-R upregulating effect of oligomeric IgD. In addition, monomeric IgD prevents the induction of IgD-R on T cells, which is observed after injection of Ag *in vivo*, and inhibits the early phase of priming for a secondary Ab response (Swenson et al., 1995). On the basis of these findings, we have suggested that T-B-cell interactions leading to GC formation and Ab maturation are facilitated by the presence of cross-linked IgD on the B-cell surface interacting with IgD-R on $CD4^+$ helper T cells (Amin et al., 1994; Swenson et al., 1995). However, in view of the observation that GC formation does occur in $IgD^{-/-}$ mice (Nitschke et al., 1993; Roes and Rajewsky, 1993) as well as in mice treated with anti-IgD from birth (Jacobson et al., 1981), such an interaction is clearly not required for GC formation.

With respect to T cells, older observations have established that GC formation is T-dependent (de Sousa and Pritchard, 1974; Jacobson et al., 1974; Stedra and Cerny, 1994), but the nature of the T cells required has not been established. It has become clear from recent studies by Fuller et al. (1993) and by Zheng et al. (1994) that a large portion of the $CD4^+$ T cells found in newly produced GCs are specific for the Ag that induced those GCs, as judged by the $V\beta$ and $V\alpha$ used to make up their TCR. It has recently been reported, in agreement with our own unpublished observations, that $TCR\beta^{-/-}$ mice fail to produce GCs in response to most immunization procedures (Dianda et al., 1996). Nevertheless, Wen et al. (1996) have obtained GC production in SCID mice receiving B cells from $TCR\beta\delta^{-/-}$ mice and $\gamma\delta$ T cells from a T-cell line of Th2 phenotype ($IL\text{-}4^+$, $IFN\text{-}\gamma^-$) that could express CD40L on its suface. In contrast to the paucity of GC production in $V\beta^{-/-}$ mice, however, GCs are frequently observed in $V\alpha^{-/-}$ mice in association with $CD4^+$ T cells bearing a variety of $V\beta$s but no $V\alpha$ on their surface (Dianda et al., 1996). The nature of the antigens recognized by these abnormal T cells needs further clarification.

With respect to the role of individual cytokines, the importance of IL-4 in the production of GCs was recently suggested by the reduced numbers of GCs in gut-associated lymphoid tissue from $IL\text{-}4^{-/-}$ mice (Vajdy et al., 1995; L. Rizzo, W. J. Simmons, and G. J. Thorbecke, unpublished observations). However, these same studies clearly showed that GCs are produced in $IL\text{-}4^{-/-}$ mice and are therefore not totally dependent on IL-4. On the other hand, a stringent requirement for the production of LT-$\alpha$ and/or its

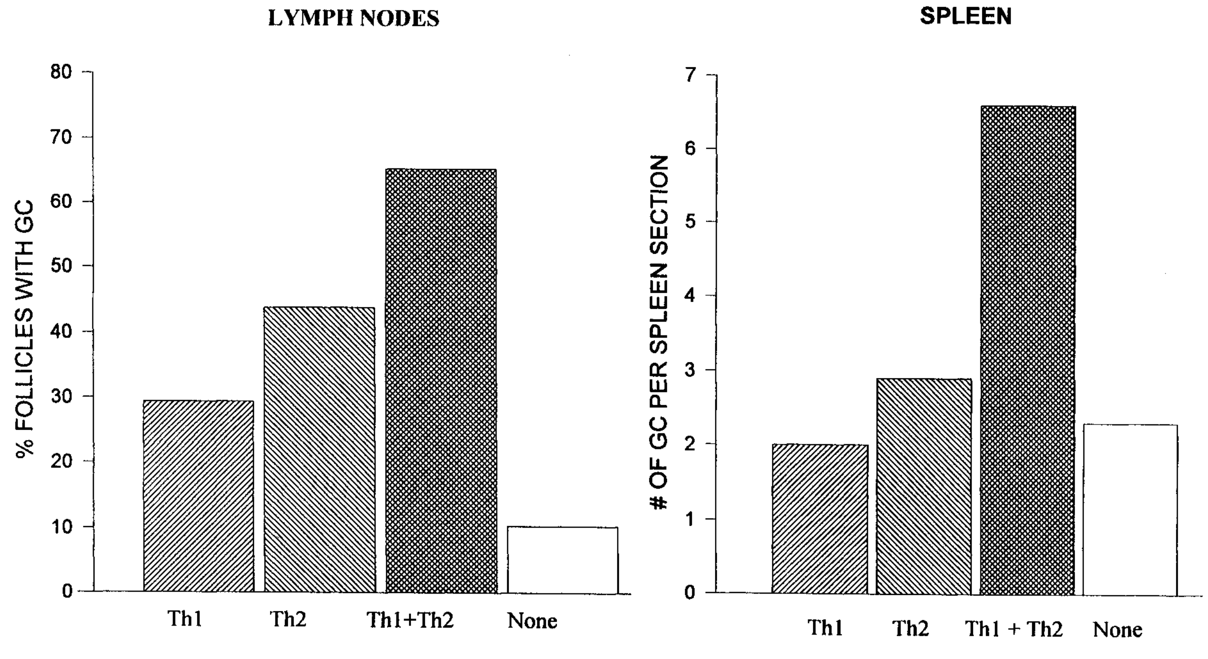

FIGURE 2 Effect of KLH-specific Thl and Th2 clones on germinal center formation to TNP-KLH in nu/nu BALB/c mice. Mice received i.v. and s.c. (in the front feet) injections of $5 \times 10^6$ cloned T cells (Thl clone D3 or LV3M and Th2 clone DC10). TNP-KLH was injected in the front feet in complete Freund's adjuvant 4 hr before the cells. Mice were boosted with TNP-KLH in saline on day 10 and killed on day 15. On the left, GCs expressed as the percentages of follicles in brachial lymph nodes exhibiting GCs; on the right is the number of GCs per splenic cross-section. Evaluation of GC numbers was performed on sections stained with PNA-peroxidase followed by counterstaining with methyl green. Data are adapted from Secord et al., (1996).

receptor TNF-RI in GC formation has been reported (Matsumoto et al., 1996), but it is not clear whether this is due to a requirement for a secreted product or for cell-surface interactions involving these molecules.

We have now asked the question whether Ag-specific CD4+ $\alpha\beta$ T-cell clones with typical Th1 or Th2 cytokine profiles are all capable of providing help for GC production (Secord et al., in press). Well-characterized KLH-specific BALB/c T-cell clones were injected i.v. and into the front foot pads of BALB/c nu/nu recipients around the same time as the Ag (TNP-KLH). The overall results (Figure 2) indicate that Th1 clones are not very capable of providing help for GC formation, whereas Th2 cells alone are capable. The most surprising result of these studies, however, is that the two clones together are more effective than either clone alone. At the previous GC meeting, we reported that IFN-$\gamma$ + IL-5 synergized in supporting B-cell CFU production in soft agar by GC B cells in response to stimulation with LPS and dextran sulfate, whereas IFN-$\gamma$ completely abolished the IL-5-induced CFU formation by peritoneal B cells (Tsiagbe et al., 1992, 1994). Thus, the results in Fig. 2 indicate that positive interaction between Th1- and Th2-derived cytokines may not only be important for proliferation of GC cells *in vitro*, but also for GC proliferation in lymph nodes and spleen *in vivo*. Another possible contributory factor to the additive effect could be that the

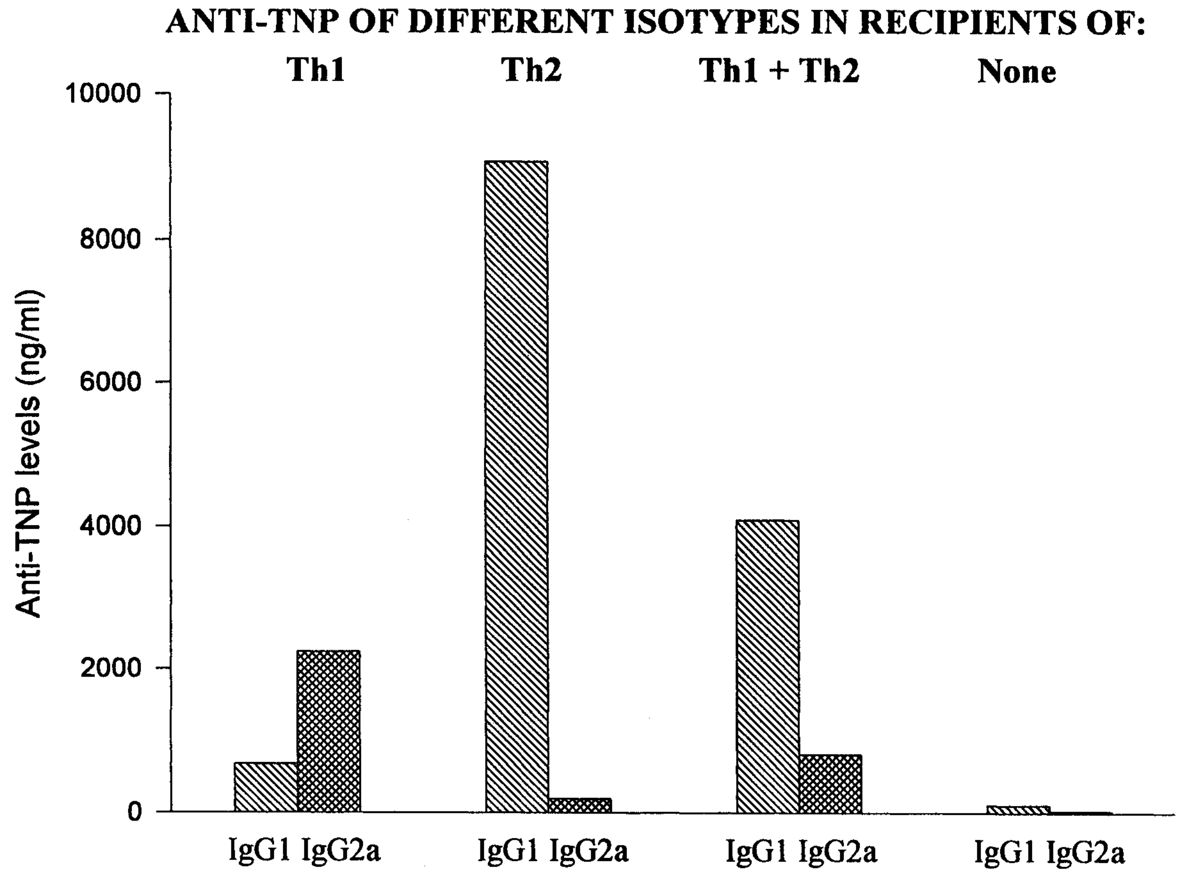

FIGURE 3 Effect of KLH-specific Th1 and Th2 clones on antibody formation to TNP-KLH in nu/nu BALB/c mice. The same mice as shown in Fig. 2 were bled on day 15 and their sera were analyzed by ELISA for anti-TNP of various isotypes. Data are adapted from Secord et al., (1996).

cotransfer of Th1 and Th2 T-cell clones may result in increased functional survival of both subsets (Rizzo et al., 1995).

The effect on the isotype distribution of the anti-TNP produced in the recipients shows a different pattern (Figure 3). Typical Th1-type helper effects are obtained with the Th1 clone, that is, high IgG2a and low IgG1, and typical Th2 helper effects with the Th2 clone, that is, high IgG1 and low IgG2a, whereas the two clones combined gave intermediate levels for both isotypes. With respect to the total number of anti-TNP-producing isotype switched cells the effect of the two clones may have been additive, but with respect to the kind of isotype produced, their effects appear antagonistic. It should be noted, however, that the cotransfer of both Th1 and Th2 clones resulted in increased production of IgA anti-TNP (data not shown).

The other question about GC T cells to which we have at this time no answer concerns the role of the NK-like $CD4^+$ T cells that occur in human GCs, the $CD57^+$ T cells (Poppema et al., 1981), and the non-H2-restricted $CD4^+$ T cells seen in follicles of the class $II^{-/-}$ mice (Cosgrove et al., 1991; Cardell et al., 1995). Do the latter represent the $NK1.1^+$ $CD4^+$ T cells that are CD-1-restricted (Bendelac et al., 1995), and if so, where is the CD-1 expression in the B-cell follicles and/or GCs? Since the $\alpha\beta$-TCR repertoire of these T cells is quite restricted, it is not likely that these T cells are responding to the Ag inducing the GC. In view of the recent demonstration that the murine $NK1.1^+CD4^+$ T cells are an important source of IL-4 (Yoshimoto et al., 1994; Emoto et al., 1995), whereas the human $CD57^+CD4^+$ T cells, isolated from tonsils, contain mRNA for IL-4 (Butch et al., 1993), it seems possible that interaction between these cells and Th1 cytokine-producing Ag-specific T cells under certain conditions may play an important role in the induction of GC formation during the primary response.

### *Acknowledgements*

This work was supported by Grant AG04980.

### *References*

Amin A.R., Swenson C.D. and Thorbecke G.J. (1994). Immunoregulatory effects of IgD. In Proceedings of Symposium in Immunology III, Eibl, M.M., Huber, C., Peter, H. H., Wahn, U., Eds. (New York: Springer Verlag), pp. 103-118.

Baine Y., Ponzio N.M. and Thorbecke G.J. (1981). Transfer of memory cells into antigen-pretreated hosts. II. Influence of localized antigen on the migration of specific memory B cells. Eur. J. Immunol. **11**:990-996.

Bendelac A., Lantz O., Quimby M.E., Yewdell J.W., Bennink J.R. and Brutkiewicz R.A. (1995). CD1 recognition by mouse $NK1^+$ T lymphocytes. Science **268**:863-865.

Butch A.W., Chung G.-H., Hoffmann J.W. and Nahm M.H. (1993). Cytokine expression by germinal center cells. J. Immunol. **150**:39-47.

Cardell S., Tangri S., Chan S., Kronenberg M., Benoist C. and Mathis D. (1995). CD1-restricted CD4+ T cells in major histocompatibility complex class II-deficient mice. J. Exp. Med. **182**:993-1004.

Coico R.F., Bhogal B.S. and Thorbecke G.J. (1983). Relationship of germinal centers in lymphoid tissue to immunologic memory VI. Transfer of B cell memory with lymph node cells fractionated according to their receptors for peanut agglutinin. J. Immunol. **131**:2254-2257.

Cosgrove D., Gray D., Dierich A., Kaufman J., Lemeur M., Benoist C. and Mathis D. (1991). Mice lacking MHC Class II molecules. Cell **66**:1051-1066.

De Sousa M. and Pritchard H. (1974). The cellular basis of immunological recovery in nude mice after thymus grafting. Immunology **26**:769-776.

Dianda L., Gulbranson-Judge A., Pao W., Hayday A.C., MacLennan I.C.M. and Owen M.J. (1996). Germinal center formation in mice lacing $\alpha\beta$T cells. Eur. J. Immunol. **26**:1603-1607.

Emoto M., Emoto Y. and Kaufmann S.H.E. (1995). IL-4 producing CD4+ TCR alpha-$\beta^{int}$ liver lymphocytes: Influence of thymus, $\beta_2$-microglobulin and NK1.1 expression. Intern. Immunol. **7**:1729-1739.

Fuller K.A., Kanagawa O. and Nahm M.H. (1993). T cells within germinal centers are specific for the immunizing antigen. J. Immunol. **151**:4501-4512.

Glimstedt G. (1936). Bakterienfreie Meerschweinchen. Acta Pathol. Microbiol. Scand. Suppl. **30**:-------.

Jacobson E.B., Baine Y., Chen Y.-W., Dhabhar P.J., Flotte T., O'Neil M.J., Pernis B., Siskind G.W., Tonda P. and Thorbecke G.J. (1981). Physiology of IgD. I. Compensatory phenomena in B-lymphocyte activation in mice treated with anti-IgD antibodies. J. Exp. Med. **154**:318-332.

Jacobson E.B., Caporale L.H. and Thorbecke G.J. (1974). Effect of thymus cell injections on germinal center formation in lymphoid tissues of nude (thymusless) mice. Cell. Immunol. **13**:416-430.

Kapasi Z.F. Burton G.F., Shultz L.D., Tew J.G. and Szakal A.K. (1993). Induction of functional follicular dendritic cell development in severe combined immunodeficiency mice. Influence of B and T cells. J. Immunol. **150**:2648-2658.

Linton P.J., Lai L., Lo D., Thorbecke G.J. and Klinman N.R. (1992). Among naive precursor cell subpopulations only progenitors of memory B cells originate germinal centers. Eur. J. Immunol. **22**:1293-1297.

Matsumoto M., Mariathasan S., Nahm M.H., Baranyay F., Peschon J.J. and Chaplin D.D. (1996). Role of lymphotoxin and the type I TNF receptor in the formation of germinal centers. Science **271**:1289-1291.

Nitschke L., Kosco M.H., Kohler G. and Lamers M.C. (1993). Immunoglobulin D-deficient mice can mount normal immune responses to thymus-independent and -dependent antigens. Proc. Natl. Acad. Sci. USA **90**:1887-1891.

Ponzio N.M., Tsiagbe V.K. and Thorbecke G.J. (1996). Superantigens related to B cell hyperplasia. In Springer Seminars in Immunopathology, Vol. 17 (New York: Springer-Verlag), pp. 285-306.

Poppema S., Bahn A.K., Reinherz E.L., McCluskey R.T. and Schlossman S.F. (1981). Distribution of T-cell subsets in human lymph nodes. J. Exp. Med. **153**:30-41.

Rizzo L.V., DeKruyff R.H., Umetsu D.T. and Caspi R.R. (1995). Regulation of the interaction between Th1 and Th2 cell clones to provide help for antibody production in vivo. Eur. J. Immunol. **25**:708-716.

Roes J. and Rajewsky K. (1993). Immunoglobulin D (IgD)-deficient mice reveal an auxiliary receptor function for IgD in antigen-mediated recruitment of B cells. J. Exp. Med. **177**:45-55.

Secord E.A., Edington J.M. and Thorbecke G.J. (1995). The E mu-*bcl-2* transgene enhances antigen-induced germinal center formation in both BALB/c and SJL mice, but causes age-dependent germinal center hyperplasia only in the lymphoma prone SJL strain. Amer. J. Pathol. **147**:422-433.

Secord E.A., Rizzo L.V., Barroso E.W.S., Umetsu D.T., Thorbecke G.J. and DeKruyff R.H. (1996). Reconstitution of germinal center formation in nude mice with Th1 and Th2 clones. Cell. Immunol. **174**:173–179.

Stedra J. and Cerny J. (1994). Distinct pathways of B cell differentiation. I. Residual T cells in athymic mice support the development of splenic germinal centers and B cell memory without an induction of antibody. J. Immunol. **152**:1718-1726.

Swenson C.D., Rizinashvili E., Amin A.R. and Thorbecke G.J. (1995). Oligomeric IgD augments and monomeric IgD inhibits the generation of IgG memory antibody responses in normal, but not in IgD-deficient mice J. Immunol. **154**:653-663.

Swenson C.D., van Vollenhoven R.F., Xue B., Siskind G.W., Thorbecke G.J. and Coico R.F. (1988). Physiology of IgD. IX. Effect of IgD on immunoglobulin production in young and old mice. Eur. J. Immunol. **18**:13-20.

Thorbecke G.J. (1959). Some histological and functional aspects of lymphoid tissue in germfree animals. I. Morphological studies. Ann. N. Y. Acad. Sci. **78**:237-246.

Thorbecke G.J., Gordon H.A., Wostman B., Wagner M. and Reyniers J.A. (1957). Lymphoid tissue and serum gamma globulin in young germfree chickens. J. Infect. Dis. **101**:237-251.

Tsiagbe V.K., Chickramane S., Amin A.R., Edington J.M. and Thorbecke G.J. (1994). Cytokine responsiveness of germinal center B cells. In Lymphoid Tissues and Germinal Centers, Heinen, E., Ed. (New York: Plenum), pp. 207-212.

Tsiagbe V.K., Nicknam M.H., Fattah D. and Thorbecke G.J. (1992). IL-5 responsive subsets among normal and lymphomatous murine B cells. Ann. N. Y. Acad. Sci. **651**:270-273.

Tsiagbe V.K., Yoshimoto T., Asakawa J., Cho S.Y., Meruelo D. and Thorbecke G.J. (1993). Linkage of superantigen-like stimulation of syngeneic T cells in a mouse model of follicular center B cell lymphoma to transcription of endogenous mammary tumor virus. EMBO J. **12**:2313-2320.

Vajdy M., Kosco-Vilbois M.H., Kopf M., Kohler G. and Lycke N. (1995). Impaired mucosal immune responses in interleukin-4-targeted mice. J. Exp. Med. **1814**:41-53.

Wen L., Pao W., Wong F.S., Peng Q., Craft J., Zheng B., Kelsoe G., Dianda L., Owen M.J. and Hayday A.C. (1996). Germinal center formation, immunoglobulin class switching and autoantibody production driven by "non alpha/beta" T cells. J. Exp. Med. **183**:2271-2282.

Yoshimoto T. and Paul W.E. (1994). $CD4^+$, $NK1.1^+$ T cells promptly produce interleukin 4 in response to in vivo challenge with anti-CD3. J. Exp. Med. **179**:1285-1295.

Zheng B., Xue W. and Kelsoe G. (1994). Locus-specific somatic hypermutation in germinal centre T cells. Nature **372**:556-559.

